# An open-label cluster randomised trial to evaluate the effectiveness of a counselling intervention on linkage to care among HIV-infected patients in Uganda: Study design

**DOI:** 10.1016/j.conctc.2016.12.003

**Published:** 2016-12-10

**Authors:** Eugene Ruzagira, Kathy Baisley, Anatoli Kamali, Heiner Grosskurth

**Affiliations:** a London School of Hygiene and Tropical Medicine, London, UK; b MRC/UVRI Uganda Research Unit on AIDS, Entebbe, Uganda; c International AIDS Vaccine Initiative, NY, USA

**Keywords:** HIV infection, Counselling, Testing, Home-based, Linkage, Care, Uganda

## Abstract

**Introduction:**

Home-based HIV counselling & testing (HBHCT) is highly acceptable and has the potential to increase HIV testing uptake in sub-Saharan Africa. However, data are lacking on strategies that can effectively link HIV-positive individuals identified through HBHCT to care. This trial was designed to assess the effectiveness of two brief home-based counselling sessions on linkage to care, provided subsequent to referral for care among HIV-positive patients identified through HBHCT in a rural community in Masaka district, Uganda.

**Methods:**

28 communities (clusters) were randomly allocated to control (referral only) and intervention (referral and follow-up counselling) arms (n = 14 clusters/arm). Randomisation was stratified on distance from the district capital (≤10 km vs > 10 km) and cluster size (larger single village vs combined small villages), and restricted to ensure balance on selected cluster characteristics. A list of possible allocations was generated and one randomly selected at a public ceremony. HBHCT is being offered to all adults (≥18 years), and HIV-positive individuals not yet in care are eligible for enrolment. The intervention is provided at one and two months post-enrolment. Primary outcomes, assessed 6 months after enrolment, are: the proportion of individuals linking to HIV care within 6 months of HIV diagnosis and time to linkage. The primary analysis will be based on individual-level data.

**Discussion:**

This study will provide evidence on the impact of a counselling intervention on linkage to care among adults identified with HIV infection through HBHCT. Interpretation of the trial outcomes will be aided by results from an on-going qualitative sub-study.

## 1 Introduction

Access to anti-retroviral therapy (ART) in sub-Saharan Africa has expanded considerably but AIDS-related mortality remains high [1]. A major cause of mortality is the late presentation of patients for care [2], [3]. Early ART initiation depends on early HIV diagnosis through HIV counselling and testing (HCT) and prompt linkage to care [4]. HCT is essential in expanding HIV prevention and treatment services [5], [6], but its uptake in sub-Saharan Africa remains low [7]. For instance, up to 74% of men and 58% of women in the region have never been tested for HIV [8].

In sub-Saharan Africa, home-based HIV testing and counselling (HBHCT) has the potential to substantially increase people's awareness of their HIV status [9]. HBHCT is highly acceptable [10], [11], [12], cost-effective at reaching previously untested persons compared with other HCT models [13], promotes equitable access of services [14] and may promote couples HCT [15], [16] and prevention of mother-to-child HIV transmission [16]. Additionally, HBHCT facilitates early HIV diagnosis and may promote early linkage to care [16]. However few studies have reported data on linkage to HIV care after HBHCT [17], [18]. Unfortunately, these studies suggest that without additional strategies to facilitate linkage, less than half of HIV-positive persons identified through HBHCT link to care [18], [19]. In contrast, HBHCT studies in which such linkage strategies were used showed an increase of linkage of up to 90% [20], [21], [22], [23], [24], [25]. However, most of these were uncontrolled observational studies. There is a need for rigorous evaluation of promising linkage strategies, using randomised controlled trials in order to identify the most effective approach.

Follow-up counselling after HIV diagnosis and referral is one of the strategies that has been utilised to facilitate linkage to care [21], [24], [25]. Counselling may mitigate psychosocial barriers of linkage to care [26]. For instance, counselling facilitates disclosure of HIV positive status [27]. In turn, disclosure makes it possible for the patient to receive psychosocial support, a key facilitator of linkage to care [28]. Follow-up counselling may also be used to provide information about ART, other care services and encourage linkage to care [29]. It is also a relatively simple strategy that may be delivered through non-medical personnel [30], [31], which makes it feasible also in low resource settings. We describe an ongoing cluster randomised controlled trial of follow-up counselling after referral to HIV care, compared to referral only, among individuals diagnosed with HIV infection through HBHCT in Masaka district, Uganda.

### 1.1 Study objectives and outcomes

The objective of the study is to determine the effectiveness of follow-up counselling on linkage to HIV care, defined as active registration with an HIV clinic. Primary outcomes are: the proportion of individuals linking to care within 6 months of HIV diagnosis through HBHCT, and time between HIV diagnosis and linkage to care. Secondary outcomes include time between HIV diagnosis and receipt of CD4 cell count test results, time between HIV diagnosis and ART initiation, and the proportion of participants who report adherence to daily cotrimoxazole prophylaxis 6 months after HIV diagnosis. A further secondary outcome is the proportion of HIV-negative participants who agree to repeat HIV testing 6 months after HBHCT.

## 2 Methods

### 2.1 Study design and population

#### 2.1.1 HIV-positive participants

The study is an open-label cluster randomised controlled trial being conducted in three rural sub-counties in Masaka district, Uganda. It is based at the MRC/UVRI Unit station in Masaka. The study population consists of newly and previously diagnosed HIV-positive adults (≥18 years) identified through HBHCT who are not in care and are willing to provide informed consent and receive follow-up counselling at home. Exclusion criteria include: previous/current receipt of HIV care, on-going participation in other health-related research, planned change of residence in the next 6 months, and inability to provide informed consent.

#### 2.1.2 HIV-negative participants

HIV-negative adults (≥18 years) who are willing to provide informed consent, receive follow-up counselling at home, and have no intention of changing residence in the next 6 months are recruited from each randomised community primarily to reduce the possibility of revealing the HIV positive status of the main trial participants. Data from these participants will also be used to investigate whether follow-up counselling increases the uptake of repeat HIV testing.

### 2.2 Identification and selection of clusters

**Fig. 1:**
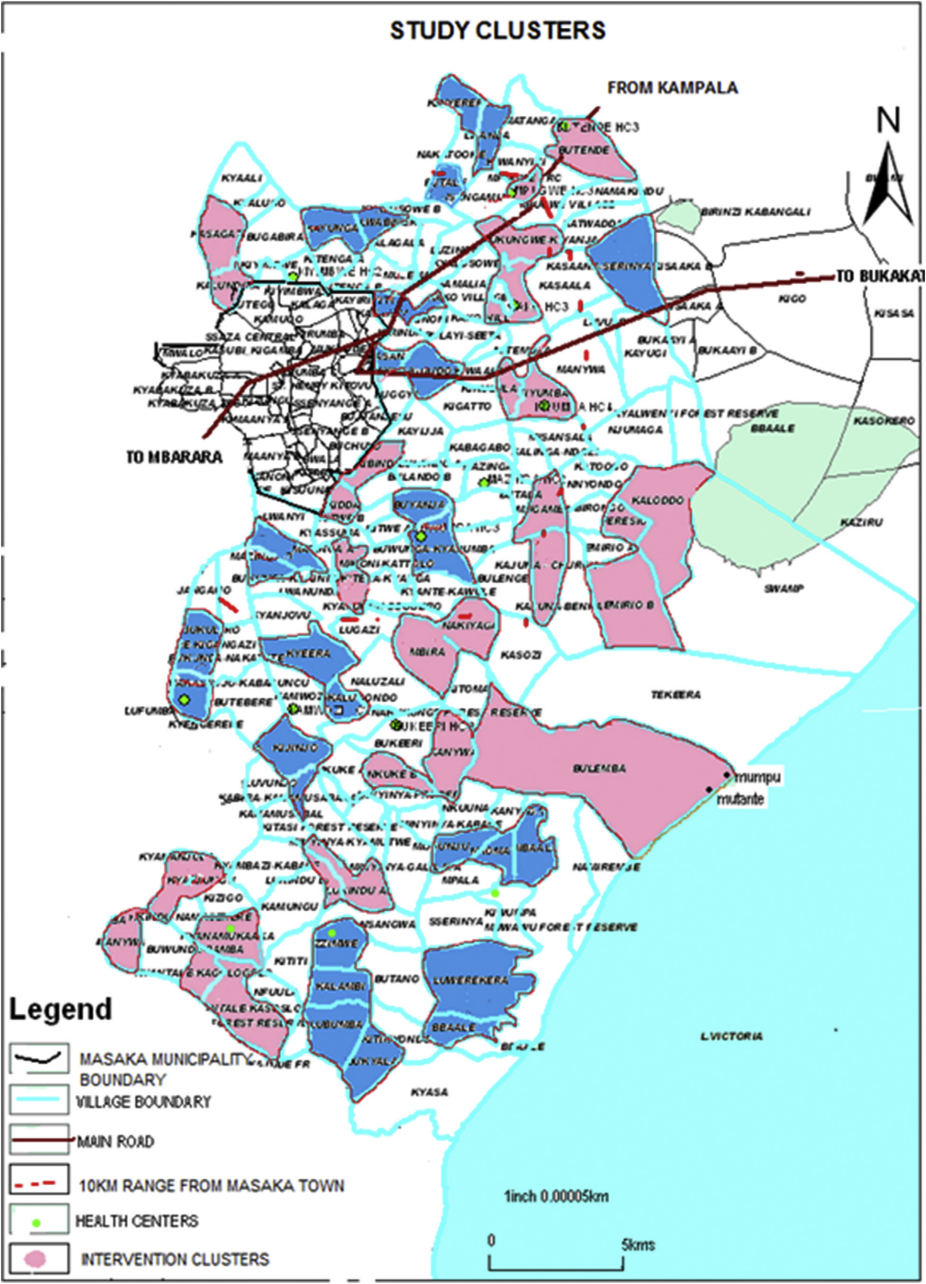
Study clusters.

Clusters comprised one or more villages i.e. the smallest administrative areas. All villages (n = 158) in the study area were mapped (Fig. 1) and the number of adults in each village obtained (range: 50–1500). To ensure a reasonable number of eligible participants in each cluster, we combined small (<400 adults) villages with adjacent villages into larger clusters of at least 400 adults. Therefore, a cluster was defined as a village or a set of villages with at least 400 adults. Clusters were separated by a buffer zone of at least one non-participating village to minimise the risk of contamination.

### 2.3 Sample size estimates

Sample size was calculated to ensure adequate power to address the hypothesis that follow-up counselling would increase the proportion of individuals that link to care. Based on previous studies in the area [32], [33], we assumed an adult HIV prevalence of 10%. The 2011 Uganda national HIV sero-prevalence survey (Masaka district was included in the survey) found that 60% of HIV-positive adults are unaware of their HIV status and are therefore not in care [33]. However, anticipating increased HCT coverage since the survey, we adjusted this figure to 40%. Based on these figures and the population estimates from the mapping data (harmonic mean of 525 adults in each cluster), we estimated that the harmonic mean number of HIV-positive persons who would not be in care in a cluster was 21 (i.e. 525 × 0.10 × 0.40). After further adjusting for persons who would be excluded due to ineligibility or refusal to participate (10%) as well as loss to follow-up (10%) after enrolment, we estimated that the harmonic mean number of individuals completing the study in each cluster would be 17.

We assumed a between cluster coefficient of variation, *k,* of 0.25. This was based on past studies of linkage to HIV care [22], [34], [35] that were conducted in settings similar to Masaka in which *k* ranged from 0.12 to 0.33. We assumed linkage of 35% in the control arm based on findings from earlier HBHCT studies [36], [37], [38], [39]. The estimated intervention effect was based on findings from HBHCT studies that have used follow-up counselling to facilitate linkage to care in Uganda [21], [22]. Using methods for stratified cluster randomised trials [40], and assuming a *k* of 0.25 and a harmonic mean of 17 participants completing the study in each cluster, we estimated that 11 clusters per arm would be required to have 90% power to detect an increase in linkage to care from 35% in the control arm to 60% in the intervention arm as statistically significant (p < 0.05). This sample size would also give us 95% power to detect a hazard ratio of 1.7 for the effect of the intervention on time to linkage, or 80% power to detect a hazard ratio of 1.5 [41].

After completing enrolment in the first seven clusters, we observed that 12% (419/3546) of registered resident adults from the mapping exercise were not found at home and could not be contacted in spite of repeated attempts. Of those found at home, 11% (358/3127) declined to have HBHCT. Furthermore, among HIV positive persons, there was a higher than expected level of engagement in HIV care: only 26% (75/294) of those who tested positive in HBHCT were not in care. As a result, the number of participants enrolled per cluster was much lower (harmonic mean < 10) than anticipated. Based on these preliminary findings, the number of clusters was increased to 28 (14 per arm) with an expected harmonic mean of 7 participants completing the study in each cluster; this would allow for detection of the same difference in proportions with a power of 83%, or a hazard ratio of 1.7 with a power of 85%.

### 2.4 Randomisation of clusters

We used a combination of stratification and restriction in order to minimise between-cluster variation and achieve overall balance between study arms with regard to key variables. First, randomisation was stratified on distance (≤10 km or >10 km) from the Masaka district capital, as this may influence accessibility to HIV care, and on cluster make-up, i.e. whether the cluster was composed of a single large village or several small villages: 70,560 possible allocations (possible ways in which clusters could be randomised to intervention or control arms) were generated under the stratified design. Restricted randomisation was then applied to achieve balance on the following covariates: cluster size; presence of a trading centre; location along a major road; lakeshore location; and presence of an HIV clinic within 5 km. The tolerance thresholds for balance were defined through an iterative process in which different thresholds were tried and the number and the validity of the acceptable allocations examined. Of the possible allocations under the stratified design, 28,932 (41%) were found to satisfy the additional balance criteria after restriction. Of these, a list of 1000 acceptable allocations was randomly generated from which one was selected at public randomisation ceremony. Each allocation had a unique running number.

#### 2.4.1 Public randomisation ceremony

The ceremony was held on 13th March 2015 and attended by 1–2 leaders from each of the villages. Three sacks each containing 10 balls labelled 0 to 9 were prepared, representing allocations 001 to 999, and 000 representing 1000. The principle underlying the random selection procedure was carefully explained in simple words. Three community leaders, selected by their peers, were then invited and one after the other each asked to draw one ball from one sack (3 balls total) thus generating a 3-digit number, corresponding to the allocation running number on the list. The six clusters added later were treated as a separate stratum. Possible allocations for these clusters were generated using the same procedure described above and one allocation selected at a second public randomisation ceremony on 30th June 2015.

### 2.5 Study interventions

Participants in the control and intervention clusters receive HBHCT and a written referral to an HIV care clinic of their choice if found to be HIV-positive. In addition, participants in the intervention clusters receive two follow-up counselling sessions at their home, delivered within 2 months after testing. Each session lasts about 45 min. The counselling intervention is designed to address common previously described psychosocial barriers of linkage to HIV care such as stigma [42], [43], [44], [45], non-disclosure of HIV status [34], [46], [47], denial of HIV diagnosis [43], [45], [47], misconceptions about antiretroviral drugs (ARV) [26], [30], [45], and fear of their side effects [26], [30], lack of partner [45], [48] and other social support [34], [47], [49], and misconceptions about ART provider practices [45], [49]. The content of the counselling intervention includes: a discussion of the individual's acceptance of HIV diagnosis, experience of stigma, plans to seek care and support services, importance of HIV status disclosure and psychosocial support for linkage to and retention in care; and provision of information about local HIV care services, ARVs, and rationale for early linkage to care. In keeping with previous observational studies of linkage to care interventions [21], [22], [25], [29], follow-up counselling in our study is conducted at one and two months after HBHCT. HIV-negative participants in the intervention arm are offered home-based risk reduction counselling and encouraged to seek regular HIV testing.

### 2.6 Study procedures

A summary of the study procedures is provided in Table 1.

**Table 1:** Study procedures.

Procedure	−3 months	−1 week	Month 0	Month 1	Month 2	Month 6	Month 6 + 1 week
Mapping & identification of clusters	X						
Community mobilisation^a^	X	X					
Recruitment and training of staff	X						
Set up of referral tracking and linkage verification procedures	X						
Census & HBHCT			X				
Study information & informed consent			X				
Eligibility screening & enrolment			X				
Collection of socio-demographic data			X				
Referral for care			X				
Follow-up counselling (Intervention arm)				X	X		
Collection of outcome data (participant interview)						X	
Collection of outcome data (review of HIV clinic records)						X	
Sample collection for CD4 cell count testing^b^						X	
Sample collection for repeat HIV testing						X	
Delivery of CD4 cell count and repeat HIV test results^c^							X

a Community mobilisation activities are conducted up to one week before onset of HBHCT in each cluster.

b CD4 count testing is done for participants who have not had a CD4 count test or are not aware of the result.

c Testing is conducted at the MRC/UVRI in Masaka town.

#### 2.6.1 Preparatory phase

Mapping of the study area, community mobilisation meetings, staff recruitment and training, setting up of referral tracking and linkage verification procedures with HIV care providers were conducted in the three months prior to study initiation. In each cluster, additional community mobilisation is conducted one week prior to HBHCT.

#### 2.6.2 Screening and enrolment

Accompanied by a community guide, study counsellors visit all the households in the randomised clusters, enumerate all resident adults aged ≥18 years and offer them HBHCT. HCT is conducted in accordance with national guidelines [50]. Married/cohabiting individuals are given the option of couple or individualised HCT. Identified HIV-positive individuals who are not yet in care are given a written referral letter (a copy is retained for the study file) to take to their preferred HIV care provider for initiation of cotrimoxazole prophylaxis, CD4 count testing (results available within 4 weeks), and ART initiation if eligible under national guidelines (CD4 <500 cells/μL). Additionally, they are given detailed information about the study and invited to consent and participate. After obtaining consent, the individual's eligibility for the study is assessed, he/she is enrolled if eligible, and informed of his/her cluster allocation. A questionnaire is then administered to collect data on socio-demographic characteristics and history of previous HIV testing history. Households in which some or all adults are not at home are revisited at least two more times.

Once 3–4 HIV-positive participants are enrolled, enrolment is also offered to the first consenting person that tests HIV negative in the next household. We expect to recruit at least 84 HIV-negative participants.

#### 2.6.3 Participant follow-up

There are four post-enrolment home-based follow-up visits.

• Months 1 and 2 (intervention arm only): to provide follow-up counselling.
• Month 6: to collect data on linkage to care and other outcomes, perform CD4 cell count testing for participants who have not had a CD4 count test at an HIV clinic or are not aware of the result, and repeat HCT for HIV-negative participants. CD4 cell count and repeat rapid HIV tests are conducted at the MRC/UVRI laboratory in Masaka town. Repeat rapid HIV tests are conducted at the laboratory instead of participants' homes in order to make the procedures for HIV-positive and HIV-negative participants as similar as possible and thus minimise the risk of revealing participants' HIV status.
• Month 6 + 1 week: to provide CD4 cell count or repeat HIV test results.

Participants not found at home at any of the post-enrolment visits are revisited at least two more times.

#### 2.6.4 Collection of outcome data

Outcome data are collected using two methods: Firstly, a questionnaire is administered by the counsellor to collect information on whether and when the participant linked to care, provided a blood sample for CD4 cell count testing and received the results, was informed of his/her ART eligibility and initiated ART, initiated cotrimoxazole prophylaxis and whether he/she adhered to cotrimoxazole prophylaxis. Good adherence to cotrimoxazole prophylaxis is defined as self-report of having taken >80% (≥25) of 30 doses in the past month. Secondly, clinic records are reviewed, looking for a participant's referral letter and medical forms, and pre-ART and/or ART register books to verify self-reported linkage and other outcomes. Participants whose records are not found at the HIV clinic are re-contacted, informed of the verification outcome, and asked to clarify if they actually registered for care or not. Except for adherence to cotrimoxazole, which is not recorded in the clinic records, only verified data will be used for the analysis of primary and secondary outcomes.

#### 2.6.5 Data management

Data are collected using paper-based questionnaires. Before data entry, questionnaires are checked for completeness and logical consistency. Data are double-entered and validated in Microsoft Access. Queries are run on the entered data every fortnight and reports sent to the study team for resolution. Data arising from the resolved queries are resubmitted for entry and the process is repeated until no more queries are generated.

### 2.7 Analysis plan

Analyses will be by intention to treat and based on individual-level data, since there is a reasonable number of clusters per arm and the cluster size varies considerably [51]. Random effects logistic regression and Cox regression with shared frailty will be used to estimate the effect of the intervention on the proportion of participants linking to care and time to linkage, respectively. Likelihood ratio tests will be performed for hypothesis testing. Similar methods will be used to estimate the effect of the intervention on the proportions of participants that adhere to cotrimoxazole prophylaxis and undergo repeat HIV testing, and on the time to receipt of CD4 count results, and ART initiation. The primary analysis of the intervention effect for all outcomes will be adjusted for randomisation stratum as a fixed effect. Exploratory analyses adjusting for age and sex a priori, and other characteristics that show substantial baseline imbalance, will also be carried out. Although the primary analyses will be based on individual-level approaches, analyses based on cluster-level approaches will also be performed to check the robustness of the results. Kaplan-Meier methods will be used to calculate time to linkage, receipt of CD4 count results, and ART initiation in each arm. Median and interquartile ranges for time to these outcomes will be estimated. A detailed statistical analysis plan will be prepared prior to data analysis.

## Ethical approval

The initial study protocol and subsequent amendments were approved by Uganda Virus Research Institute Research Ethics Committee (GC/127/14/12/491), the Uganda National Council for Science and Technology (HS 1732), and The London School of Hygiene and Tropical Medicine Ethics Committee (8833). Written informed consent (including consent to track referrals and review medical records for persons who link to care) is obtained from each participant before study procedures are conducted. The trial is registered at ClinicalTrials.gov (NCT02497456).

## 3 Discussion

This study is one of the few randomised trials from sub-Saharan Africa designed to evaluate the effect of an intervention to increase linkage to HIV care following HIV diagnosis through HBHCT. We are aware of five other such trials. The first is a recently completed individually randomised controlled trial from South Africa and Uganda that assessed whether community-based HIV testing (including HBHCT) with counsellor support (home-based follow-up counselling and accompanied referral) and POC CD4 cell count testing increases uptake of ART. In this trial, linkage to care was high (≥89%) across all linkage strategies used [20]. The second is a completed but not yet reported individually randomised trial from rural western Kenya aiming to assess the accuracy, feasibility and acceptability of point-of-care (POC) CD4 testing for persons found HIV positive through HBHCT, and to determine the (cost-) effectiveness of this intervention in improving linkage to care and time to ART initiation [52]. The third is an ongoing cluster randomised trial in rural Uganda to evaluate the effectiveness of linkage to care counselling in achieving HIV viral suppression and to determine intermediate outcomes (linkage, time to initiation of opportunistic infection prophylaxis, and time to initiation of ART among people testing HIV positive during HBHCT) [53]. The fourth is an ongoing household randomised trial in rural Lesotho to evaluate the effectiveness of same day home-based ART initiation after HIV diagnosis coupled with a reduced frequency of clinic follow-up visits in improving linkage to care, retention in care, and viral suppression [54]. The fifth is a planned individually randomised trial in western Kenya that will compare home and other community recruitment strategies, HIV testing strategies (self-testing, HCT in a home/mobile setting, and facility-based HCT), and different linkage strategies (referral only, text message reminder, and financial incentive) to an adaptive (SMART trial design) linkage to care intervention, among young at-risk women [55]. Primary outcomes for this trial include uptake of recruitment strategies, uptake of testing modalities, linkage to and retention in care.

A major strength of our trial is that referrals for care are tracked and medical records examined to verify self-reported linkage. Hence it is unlikely that the proportion of persons linking to care will be overestimated. Despite concerns about inadequate routine record keeping at health care facilities in Uganda [56], we have so far not encountered such a problem at HIV care clinics. All information on reported linkage collected and analysed to date have either been verified by clinical records, or have been withdrawn as false when individual patients were re-interviewed in case of discrepancies. Participants who self-report not linking to care are not tracked further, as it is unlikely that individuals would link to care but choose to report otherwise.

We randomised communities instead of individuals in order to minimise the risk of contamination between trial arms e.g. through sharing of follow-up counselling information, which would result in a dilution of intervention effects. Also, individual randomisation may not have been acceptable to participants especially if they perceive one of the interventions to be inferior to the other.

The study has some limitations. First, some individuals in the study communities may have knowledge of their cluster allocation which may influence their decision to enrol in the study. Individuals may obtain prior knowledge of cluster allocation through community leaders who attended the pre-enrolment public randomisation ceremony or from those in the same community who have enrolled before them. To minimise the effect of this potentially differential enrolment, individuals are only informed of their cluster allocation after they have consented and enrolled in the study. Additionally, within each village, participants are enrolled over 2–4 days, to minimise the time available for individuals to find out their allocation from fellow village residents. Related to this is the risk of differential loss to follow-up after enrolment e.g. because different proportions of participants may withdraw from the trial after becoming aware of their allocation. To mitigate against this, part of the eligibility criteria is “availability for the entire duration of the study and willingness to be followed up at home”. Participants who indicate that they will not be available for follow-up are excluded from the study. Second, despite the creation of buffer zones between clusters, there might still be a risk of contamination resulting from possible interaction between study participants from different trial arms. Data on the occurrence of such interaction is collected at the month-6 visit; the results will be reported along with the main trial findings.

We did not include a component to evaluate cost-effectiveness of the home-based follow-up intervention. Such evaluation may be necessary to inform policy if the intervention is found to be effective. However, given the fact that it is a relatively simple intervention, the costs of incorporating it into the routine HBHCT work are likely to be minimal.

Whereas quantitative methods such as those used in our trial are appropriate for investigating the effect of a health care intervention, a qualitative exploration of experiences, attitudes, beliefs and understandings is needed to find out why the intervention works or does not work [57], [58]. For this reason, a qualitative sub-study is being conducted in both trial arms in a sub-set of participants who linked successfully to care and others who did not in order to better understand the reasons for linkage or lack of linkage to HIV care.

This study will contribute evidence on the impact of referral plus home-based follow-up counselling on linkage to care, as compared to only referral among adults identified with HIV infection through HBHCT in Uganda. Interpretation of trial outcomes will be aided by findings from the qualitative sub-study.

## Funding

This study was jointly funded by the UK Medical Research Council (MRC) and the UK Department for International Development (DFID) under the MRC/DFID Concordat agreement and is also part of the EDCTP2 program supported by the European Union. The International AIDS Vaccine Initiative provided funds for HIV test kits. The Department of Infectious Disease Epidemiology, London School of Hygiene & Tropical MedicineLondon School of Hygiene & Tropical Medicine provided PhD support funds to cover CD4 count tests.
